# Impact of Packet Loss Rate on Quality of Compressed High Resolution Videos

**DOI:** 10.3390/s23052744

**Published:** 2023-03-02

**Authors:** Juraj Bienik, Miroslav Uhrina, Lukas Sevcik, Anna Holesova

**Affiliations:** University of Zilina, Univerzitna 1, 010 26 Zilina, Slovakia

**Keywords:** H.264/AVC, H.265/HEVC, QoE, QoS, packet loss rate, video quality

## Abstract

Video delivered over IP networks in real-time applications, which utilize RTP protocol over unreliable UDP such as videotelephony or live-streaming, is often prone to degradation caused by multiple sources. The most significant is the combined effect of video compression and its transmission over the communication channel. This paper analyzes the adverse impact of packet loss on video quality encoded with various combinations of compression parameters and resolutions. For the purposes of the research, a dataset containing 11,200 full HD and ultra HD video sequences encoded to H.264 and H.265 formats at five bit rates was compiled with a simulated packet loss rate (PLR) ranging from 0 to 1%. Objective assessment was conducted by using peak signal to noise ratio (PSNR) and Structural Similarity Index (SSIM) metrics, whereas the well-known absolute category rating (ACR) was used for subjective evaluation. Analysis of the results confirmed the presumption that video quality decreases along with the rise of packet loss rate, regardless of compression parameters. The experiments further led to a finding that the quality of sequences affected by PLR declines with increasing bit rate. Additionally, the paper includes recommendations of compression parameters for use under various network conditions.

## 1. Introduction

A high standard of living, education, and more opportunities, along with people’s demands for such better conditions, has been constantly increasing over the last decades. Naturally, this applies to expectations in all areas of life, including entertainment and technology. Much like the rest of the world, Internet service providers, as well as broadcasting companies, want to bring their customers the best possible experiences. This individual user impression, also referred to as quality of experience (QoE) is highly dependent on quality of service (QoS) parameters. In the field of video transmission, these further depend on factors such as latency, jitter, or packet loss rate (PLR). However, although such factors are easily measurable, QoE cannot be quantified so effortlessly. Nowadays, one of the most popular network services is live video streaming, which is growing on a huge scale [1,2]. However, the video delivered over an IP network in real-time applications, is often exposed to various types of quality degradation. One of the reasons is that this type of application operates on RTP over unreliable UDP. The major sources of impairments are video compression and transmission over the noisy communication channel. Of the latter, the factor which affects video quality the most is undoubtedly PLR. It is due to consider that even a small percentage of lost packets can cause a massive decline in perceived video quality. This is proven in the following sections of the paper.

This paper is focused primarily on evaluating the influence of PLR on the quality of H.264/AVC and H.265/HEVC-encoded full HD and ultra HD video sequences. The different combinations of the resolution and compression parameters were used. Objective and subjective evaluation methods, namely peak signal-to-noise ratio (PSNR), Structural Similarity Index (SSIM), and absolute category rating (ACR) were employed for video-quality quantification. Additionally, the principle of the subjective ACR method enabled further research into the combined effect of PLR and compression parameters on perceived video quality. As a result, this paper also suggests the most suitable combination of resolution, bit rate, and codec for use under different conditions.

The paper is organized as follows. Section 3 illustrates the dataset compilation procedure, and briefly describes the source sequences and the process of video encoding and packet loss simulation. Section 4 deals with the acquisition and analysis of the results of objective video quality assessment. Analogously, Section 5 covers subjective video-quality evaluation. In addition, the analysis of the subjective results is extended by the calculation of the correlation with the objective assessment results. Moreover, further examination of the combined impact of PLR and compression on subjective video quality is described. Section 6 concludes the paper.

## 2. State of the Art

There are several research papers that deal with QoS and QoE evaluation, especially the performance assessment of various compression standards or the effect which has the transmission over noisy communication channel on the video quality. Coding structures, syntax, various tools, and settings relevant to coding effectivity were described in that paper. Human perception of compression, spatial and temporal information was examined in [3]. The authors have compiled a large-scale database of video sequences whose quality was subjectively evaluated. The coding efficiency of HEVC standard was compared with earlier codecs in [4]. The authors in [5] examine the effects of network impairment on HEVC video streaming. The impact of packet loss on the video quality and the main factors observed to be most perceived by the end-users were evaluated. In [6], a detailed, quantitative analysis of the video-quality degradation in a homogeneous HEVC video transcoder together with the analysis of the origin of these impairments and the influence of quantization step alignment on transcoding were presented. Differences between video transcoding and direct compression were described as well. The authors have also found the dependence between the quality degradation caused by transcoding and bit rate changes of the transcoded data stream.

A parametric planning model combining characteristics of the channel and the video is introduced in [7]. The video distortion due to the packet loss can be estimated by this model. Authors in [8] presented the first empirical study of the impact of loss-related errors on TV viewing engagement. They compared different platforms and delivery technologies. Video sequences and information about quality of delivery obtained from the service provider were used in the experiments. The length of the sequences, content, and connection type were compared. In [9], 16 types of metrics for the quality evaluation were compared. Packet loss was simulated in the video coding, and losses were subsequently concealed by using various error-concealment techniques. The purpose was to show that the subjective video quality cannot be predicted only from the visual quality of a frame, when some concealed error occurs. A new objective measure XLR (piXel Loss Rate) indicator was proposed in [10]. It evaluates the packet loss rate in video streaming. This method achieved comparable results with full-reference metrics and a very high correlation with MOS. Authors in [11] provided an overview of packet losses in wi-fi networks, mainly multimedia and real-time applications. A method for estimation of packet delivery ratio, which depends on the length of the packet, where longer packets are more likely to be damaged, is described in [12]. The loss of a compressed video packet results in an estimate of the expected mean square error distortion. In [13], the authors examined whether the packet loss model and primarily length of series have an impact on the accuracy of the error estimation.

The authors of [14] claimed that the highly textured video content is difficult to compress, as it is essential to achieve the compromise between bit rate and perceived video quality. Based on this, they introduced a synthetic video texture dataset that was generated by using a computer-generated imagery environment. It was named BVI-SynTex video dataset and was conformed from 196 video sequences clustered in three different texture types. It includes five-second-long FHD video scenes with a frame rate of 60 fps, and 8 bits depth.

In [15] a method for the optimal classification of the packets was proposed. They got different priority assigned when the transmission conditions became poor. The network transmits the segments with the greatest contribution toward the quality of perception if limited network conditions occur. The results showed that the proposed method can achieve a higher MOS compared to nonselective packet drop.

The purpose of this paper is not to point out the difference between QoS and QoE. These concepts, their correlation, and the derived mapping function were described in our previous paper [16]. The differences between the concepts of QoE and QoS are described in the Table 1. PLR is one of the basic QoS parameters, and the purpose of this paper is to show how this purely technical parameter can affect the perception of QoE. Thus, the main output is the mapping function of one of the QoS parameters to the metrics which represent the QoE. The selected relevant works were compared with our paper. HD video sequences based on motion in pictures were analyzed in the paper [17]. All sequences were encoded into H.264 and H.265 compression standards. The video quality was objectively measured by PSNR and SSIM metrics and subjectively assessed by using DMOS value. Compressed video files were streamed by an emulated 5G network. This network has a specific type of PLR based on different characteristics of radio channels compared with a wired metallic network. Two types of dynamic sequences (low motion and high motion) were compared. The authors did not use various sequences from the aspect of contrast. Paper [18] is focused on streamed videos based on TCP segments via a 5G network. Our paper analyzes video transmission over the metallic network with services based on RTP protocol and UDP segments. Authors in the paper [18] used more network parameters in their experiments, like bandwidth and delay. These parameters are especially critical for wireless networks, except for ISDN and low-speed xDSL networks. In paper [19], a QoE smart algorithm based on using a machine learning model is proposed. The model is based on the wireless network. It takes into account the GOP size, and technical parameters of endpoint devices (CPU, RAM, and screen size). In this manuscript, QoS parameters were fixed—280Mbps of throughput, 3 ms of two-ways packet latency, and 0.001% for packet loss and jitter. The authors describe a comparison between the traditional and adaptation approaches. Results using the proposed algorithm improved the quality. The transmission of videos via the network with various parameters of PLR was compared in our paper. The difference from our paper is that the parameters of the network are constant in the paper [19]. The mentioned parameters are changing dynamically and randomly in a real 5G radio network.

## 3. Dataset Compilation

### Selection of Source Sequences

**Figure 1:**
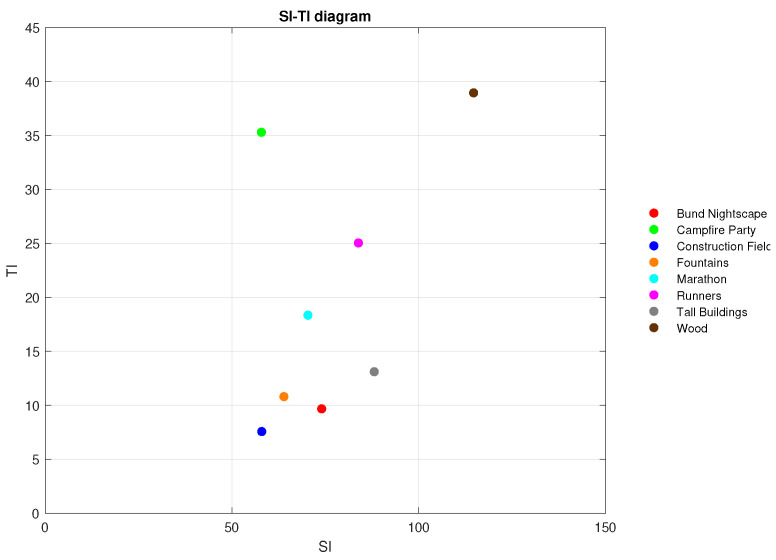
SI-TI diagram of reference sequences.

The basic requirements for reference sequences intended for further processing are uncompressed format, high bit depth, resolution and frame rate, suitable aspect ratio, and sufficient diversity in terms of scene content. Meeting all these standards, eight high-quality video sequences were selected from the Shanghai Jiao Tang University dataset [20]. Common parameters of these reference sequences are presented in Table 2. Original sequences vary in temporal (TI) and spatial perceptual information (SI) as shown in Figure 1, colors, contrast, and other features.

Spatial perceptual information (SI) indicates the amount of detail in the video frames. The equation for calculating SI, based on the Sobel filter, is defined as

(1) $$SI=max_{time}\left[std_{space}\left[Sobel\left(F_{n}\right)\right]\right],$$

where $Sobel(F_{n})$ stands for the video frame at time *n* filtered by using the Sobel filter, $std_{space}$ is the standard deviation over the pixels of the previously filtered frame, and $max_{time}$ represents the maximum spatial information value of the entire video sequence. Temporal perceptual information (TI) expresses the degree of temporal activity, i.e., the change of the corresponding pixels values in consecutive frames of the video sequence. This change is called the motion difference feature $M_{n}\left(i,j\right)$ and is defined as a function of time,

(2) $$M_{n}\left(i,j\right)=F_{n}\left(i,j\right)-F_{n-1}\left(i,j\right),$$

where i,j are the pixel coordinates in the adjacent frames *n* and n−1. The TI is then computed as

(3) $$TI=max_{time}\left[std_{space}\left[M_{n}\left(i,j\right)\right]\right],$$

where $max_{time}$ is the maximum standard deviation $std_{space}$ of the $M_{n}\left(i,j\right)$ over time.

**Figure 2:**
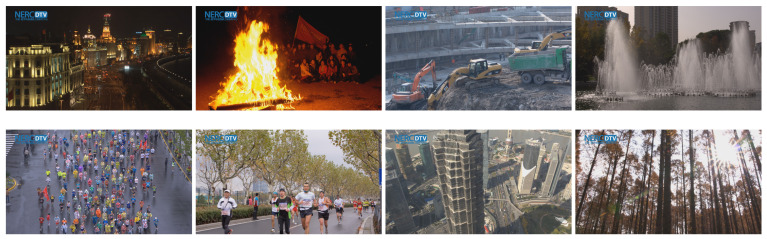
Previews of used sequences: Bund Nightscape, Campfire Party, Construction Field, Fountains (top row from the left), Marathon, Runners, Tall Buildings and Wood (bottom row from left).

Both SI and TI are calculated for the luminance component of the frames only. Higher TI indicates more motion in the scene, whereas higher SI is generated by frames with more edges and disparity in luminance [21]. Previews of the employed sequences are displayed in Figure 2, followed by a brief description of the scene content and the scenario of each video.

The Bund Nightscape is a time-lapse video sequence capturing cars driving through a night city near a river. The video was shot from a high angle with a static camera. The only sources of movement are passing cars, people in the streets, and flags hoisted on the roofs of buildings. The Campfire Party is a nighttime video sequence, picturing a group of people posing for a photograph by a campfire. The camera is almost still, except for the slight zoom in the end of the sequence. Most of the motion is generated by the blazing flames in the foreground. The Construction Field is a relatively static video sequence depicting an excavator digging a foundation pit on a construction site. Apart from this, the only other movement is caused by several people walking in the background. Fountains is a video sequence showing a large fountain with several jets situated in a small square between trees and buildings in the background. The video was recorded with a still camera; hence only the water gushing from the fountain adds dynamism to the scene. Marathon is a video sequence shot from a bird’s eye view by an almost motionless camera. The scene depicts many people dressed in colorful raincoats competing in a race on a wet asphalt road during heavy rain. Runners is a video sequence capturing dozens of people in numbered jerseys racing on a road lined with trees. Even though the scene is filmed with a static camera, its considerable dynamism is added to by the fast movement of the runners and the wind in the leaves of the trees. Tall Buildings is a video sequence, recorded from a bird’s eye perspective, that captures the tallest Shanghai skyscrapers. The camera pans steadily, revealing a view of the driving cars in the distance, which are the only moving objects in the video. Wood is a video sequence recorded in bright daylight, picturing a sunlit forest. The camera moves with a fairly swift, panning motion. It is the most complex sequence, considering the amount of detail and motion in the video.

#### Video Encoding and Packet Loss Simulation

First, all eight reference video sequences were subjected to chroma subsampling from the original YUV 4:4:4 to YUV 4:2:0 format. It is followed by a change in a bit of depth from 10 to 8 bits per channel. Afterward, these modified sequences were encoded by using two conventional standards, H.264 and H.265 at full HD and ultra HD resolutions. The bit rate ranged from 1 to 15 Mbps, employing the well-known FFmpeg software [22]. A description of codecs settings is possible to find in the Table 3. The used settings of both compression standards were the same. The target bit rate of 1 Mbps for the videos coded by H.264 could cause some artifacts which may not meet users’ expectations, but we used these extreme values due to codec quality comparison—it is well known that the highest codec efficiency is in low bit rates, especially in newer codecs, as, for instance, H.265/HEVC. The frame rate remained set at 30 fps, resulting in the fixed group of pictures (GOP) length of 15 frames. The GOP size setting was derived from the frame rate as is standard for video intended for transmission through a noisy communication channel when an unreliable protocol is used for data transfer. The GOP length actually defines the interval between two consecutive keyframes—intracoded (I) or predicted (P). Considering this fact, it seems logical that the loss of image information for more than half a second (half the frame rate value) could be unacceptable for real-time applications such as video telephony or live streaming. The number of bidirectional predicted (B) frames was set to 3, as is recommended [23] for H.264 and H.265 codecs. The structure of employed FFmpeg commands was organized as follows from the example

ffmpeg -i Marathon_1920x1080_30fps_420_8bit_YUV.yuv -vcodec libx264 -x264-params keyint=15:min-keyint=15:bframes=3:b-adapt=1:bitrate=1M:vbv-maxrate=1M:vbv-bufsize=1M Marathon_1920x1080_30fps_420_8bit_H264_1M.ts.

**Figure 3:**
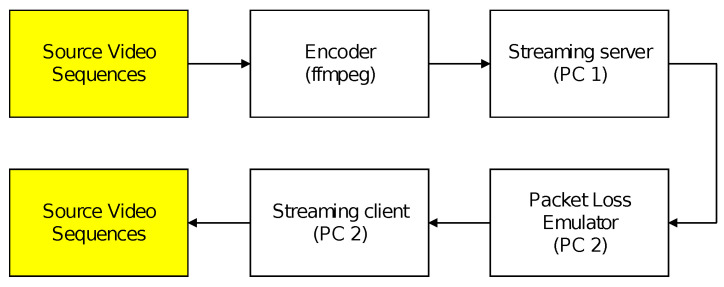
Process of video encoding and packet loss simulation.

Subsequently, a local area network consisting of a streaming server and a streaming client was assembled to serve as a transmission channel for the previously encoded sequences. To simulate a lossy network connection, Clumsy [24] software was installed on the receiving computer, which assumed the role of the streaming client. By employing this tool, a certain percentage of UDP packets from every compressed video sequence was artificially intercepted. To approximate the actual operation of IP networks, clustered packet losses were generated pseudorandomly during video transmission. Both the broadcast server and the client were represented by standard computers—laptops with gigabit LAN ports with the Windows operating system installed. The interconnection of both devices was point-to-point type by using a cat6 metallic network cable. To minimize the occurrence of errors, the Wi-Fi adapters were disabled on both computers and the Windows firewall was also disabled. As for the stream itself, the compressed data was encapsulated in a TS container. The reason for using TS is that it is still currently the most used container for multimedia stream distribution. It is not only in Ethernet networks, but also for distribution in DVB-T/DVB-T2 systems. The transport stream allows multiplexing of streams (PES and PS) that do not necessarily share a common time base for transmission in a noisy environment. Thus, its biggest advantage is robustness to transmission errors. The image data was transmitted as UDP segments by using the RTP transport protocol. RTP was chosen based on the nature of the data. The real-time data and UDP segments are usually used for transmission in broadcast networking, because there is no possibility of retransmitting failed packets. It is not like a video-on-demand service where, in contrast, TCP segments are used. The resulting packet loss rate (PLR) ranged from 0 to 1%. A block diagram illustrating the combined process of video encoding and packet loss simulation is shown in Figure 3.

Applying all possible combinations of compression parameters and PLR listed in Table 4, 1120 processed video sequences were created. To ensure higher statistical accuracy of the quality-assessment results, each video sequence was transmitted ten times over the assembled network, increasing the total number of test sequences to 11,200.

## 4. Objective Quality Evaluation

Objective quality measurement of the prepared video sequences was performed by using the MSU Video Quality Measurement Tool [25]. Two well-known objective methods, PSNR [26] and SSIM [27], were used for the evaluation; however, only the results of the SSIM metrics are presented in the paper. These showed a slightly higher correlation with the results of the subsequent subjective evaluation. The SSIM is a full-reference metric which focuses on measuring distortions in the structure of the image arising from changes in brightness, contrast, blurring of the image and so forth. This objective measure is grounded on the premise, that the human visual system is better suited to detect structural changes in the image than to identify specific errors. This can also explain the higher correlation of the SSIM quality ratings with the results of subjective quality assessment. The core principle of the SSIM consists of dividing the frames of two video sequences into areas of several pixels (depending on the size of the sliding window), in which the three components of the signal—brightness (l), contrast (c), and structure (s)—are sequentially compared. These components can be calculated by using the following formulas,

(4) $$l\left(x,y\right)=\left(2\mu_{x}\;\mu_{y}+c_{1}\right)/\left(\mu_{x}^{2}+\mu_{y}^{2}+c_{1}\right)$$

(5) $$c\left(x,y\right)=\left(2\sigma_{x}\;\sigma_{y}+c_{2}\right)/\left(\sigma_{x}^{2}+\sigma_{y}^{2}+c_{2}\right),$$

(6) $$s\left(x,y\right)=\left(\sigma_{x}y+c_{3}\right)/\left(\sigma_{x}\;\sigma_{y}+c_{3}\right),$$

where $\mu_{x}$ and $\mu_{y}$ denote the average values of the two nonnegative signals x and y, $\sigma_{x}$ and $\sigma_{y}$ are their standard deviations and $\sigma_{x}y$ expresses the covariance of these signals. The variables $c_{1}$, $c_{2}$ and $c_{3}$, defined by the following formulas, are included to stabilize the results if the denominator is close to zero,

(7) $$c_{1}={\left(K_{1}\;L\right)}^{2},$$

(8) $$c_{2}={\left(K_{2}\;L\right)}^{2},$$

(9) $$c_{3}=c_{2}/2,$$

where *L* denotes the dynamic range of pixel values, and $K_{1}$ and $K_{2}$ are low value constants.

The resulting SSIM value is determined by the weighted product of the brightness, contrast, and structure components as

(10) $$SSIM\left(x,y\right)={\left[l\left(x,y\right)\right]}^{\alpha }{\left[l\left(x,y\right)\right]}^{\beta }{\left[l\left(x,y\right)\right]}^{\gamma }.$$

By setting the weights α, β and γ to 1, the final calculation can be simplified to

(11) $$SSIM\left(x,y\right)=\frac{\left(2\mu_{x}\mu_{y}+c_{1}\right)\left({2\sigma }_{xy}+c_{2}\right)}{\left(\mu_{x}^{2}+\mu_{y}^{2}+c_{1}\right){(\sigma }_{x}^{2}+\sigma_{y}^{2}+c_{2})}.$$

The results obtained by using the SSIM metrics fall within the interval [0, 1], where 1 expresses the best quality, which can be achieved only if all the compared frames are identical. It is a symmetric method, which means that the order of the reference and degraded video sequence is insignificant. Given that the SSIM is a full-reference metrics, it would seem logical to choose an uncompressed video sequence unimpaired by transmission over the IP network as a reference. The aim of our research was to investigate the effect of PLR on quality of video in different resolutions with various compression parameters. It was not directly about the combination of PLR and effect of compression. This is a reason why the corresponding sequence with identical bit rate undegraded by network transmission was always chosen as the reference sequence for the metrics. In this way, a total of, 9600 objective measurements were performed by using the SSIM method.

### Analysis of the Results

**Figure 4:**
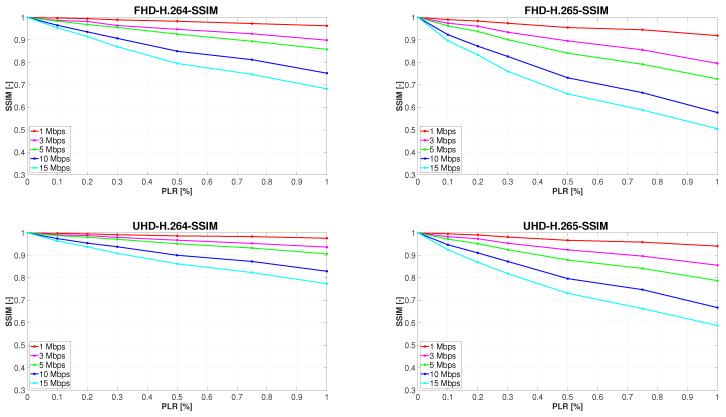
Development of objectively measured video quality with increasing PLR.

In view of the fact that the purpose of this particular research is not examining the impact of the scene content on video quality, graphs contained in the paper present only the average quality ratings. It is the same over all types of sequences, with differing content for each codec and resolution evaluated. Furthermore, it should be noted that the results of the evaluation of each sequence that was streamed 10 times were also averaged. Figure 4 outlines the development of measured video quality affected by the gradual increase in PLR, which is plotted on the *x*-axes of the graphs. The *y*-axes represent the measure of video quality evaluated by the SSIM metrics.

Because the video sequences unaffected by PLR encoded to both H.264 and H.265 in full HD and ultra HD, each bit rate of interest was used as a reference for the objective metrics. It is understandable that the quality of every sequence with 0% PLR was rated with an SSIM value of 1. Another common feature of Figure 4 is the tendency to objectively measure video quality at all bit rates to decline with the increasing PLR, as was anticipated. A full-reference objective metric such as SSIM always evaluates a video that lacks more information as of lower quality. An interesting discovery is that increasing PLR had a greater negative effect on the quality of full HD sequences than those in ultra HD resolution. Moreover, videos encoded to H.265 were rated worse on average than H.264-encoded sequences. The last and most unexpected finding is a faster decrease in the quality of sequences with a higher bit rate, which is clearly observable with all combinations of codec and resolution used. This fact, although counterintuitive because the quality of the compressed sequences with 0% PLR grows with the increasing bitrate, may have a logical explanation. For illustration, file sizes of the H.265 ultra HD Marathon video sequence at all examined bit rates are presented in Table 5. It is self-explanatory that by applying the same PLR, more bytes of data will be lost from larger files. Although the loss probability remains unchanged, it will most likely not affect the same part of the compressed sequences. It also will not affect the same number of parts of the video compressed at different bitrates, which is more important. This is due to the fact that the average size of the captured UDP packets is equal for each streamed sequence, which means that at a higher bit rate, the number of lost packets must also be larger. Therefore, most individual losses occur when video at 15 Mbps is transmitted. As the research in [28] proves, video quality degrades more significantly when several individual packets are lost than in a case when loss of the same number of consecutive packets occurs, which can be attributed to the temporal propagation of the errors. Regarding our investigation, it follows that the higher the bit rate used, the more individual or clustered packet losses will occur. Although at higher bit rates the losses are likely to affect a smaller portion of the frames, they may continue to propagate in time and space, causing more substantial video quality degradation.

## 5. Subjective Quality Evaluation

A subjective video-quality assessment was conducted to verify the findings that emerged from the analysis of the objective quality evaluation results. In addition to determining the strength of the correlation between subjective and objective measurement methods, the second reason for performing subjective tests was to monitor the combined impact of bit rate and PLR on respondents’ views of video quality. To ensure sufficient diversity of visual content while limiting the time requirements of test sessions, the four most diverse video sequences were selected for subjective quality assessment according to the SI-TI diagram, namely Campfire Party, Construction Field, Tall Buildings, and Wood. Considering that each compressed video was streamed and objectively evaluated 10 times, the sequence that achieved the median SSIM value was always chosen for the subjective tests. Bearing in mind all possible combinations of the given resolution, codec, bit rate, and packet loss rate, 560 test sequences were thus selected. The popular absolute category rating (ACR) was adopted for subjective quality assessment of the prepared video sequences. This single-stimulus method was chosen on the grounds that the test conditions are analogous to those under which a regular user watches multimedia content. The course of ACR assessment, described in ITU recommendation [21] is faster and less complicated compared to other subjective evaluation methods. In addition, the results of the assessment are reproducible quite accurately even by different groups of respondents, as proven in [29]. The test presentation comprised degraded video sequences lasting approximately 10 s, alternating with a medium gray color displayed on the screen during the evaluation period of five seconds. Respondents were instructed to rate the video quality according to the following scale divided into five levels:

- 5—Excellent
- 4—Good
- 3—Fair
- 2—Poor
- 1—Bad.

A total of 36 laymen with no visual impairment participated in the experiment, of whom 14 were women and 22 were men. The age bracket of respondents ranged from 16 to 62 years, and the average age was approximately 30 years. After the completion of the experiment, the elimination of outliers was necessary. For this purpose, a Pearson linear correlation coefficient (PLCC) was calculated between the evaluation results of all video sequences from one respondent and the average rating of each sequence, following the equation

(12) $$PLCC=\frac{\sum_{i=1}^{N}\left(X_{i}-\bar{X}\right)\left(Y_{i}-\bar{Y}\right)}{\sqrt{\sum_{i=1}^{N}{\left(X_{i}-\bar{X}\right)}^{2}}\sqrt{\sum_{i=1}^{N}{\left(Y_{i}-\bar{Y}\right)}^{2}}},$$

where *N* is a number of evaluated sequences, $X_{i}$, $Y_{i}$ are individual indexed ratings and $\bar{X}$, $\bar{Y}$ are the arithmetic means of all ratings. The threshold value of PLCC for removing a respondent’s results from further processing was set at 0.75. The correlation was examined separately on four sets of video sequences, assorted according to resolution and codec used. The number of detected outliers for each set ranged from 1 to 3 out of all 36 respondents, leaving a sufficient number of results for subsequent statistical analysis.

### 5.1. Analysis of the Results

The first step in processing the obtained subjective data was the calculation of the mean opinion score (MOS) of each evaluated video sequence. Statistical analysis of the first half of the evaluation results revealed that it is quite sufficient to inquire about the users’ opinion on the quality of video with a maximum PLR of 0.5% because after exceeding this value, the subjective quality was rated as poor at the utmost on the ACR scale. Between 0.5% and 1% PLR, the perceived quality further decreased on average by 0.28 and by a maximum of 0.45 MOS points. On that account, and for greater clarity, graphs included in this section show the development of subjectively perceived video quality with packet loss rates ranging from 0 to 0.5%.

### 5.2. Correlation with the Objective Evaluation Results

**Figure 5:**
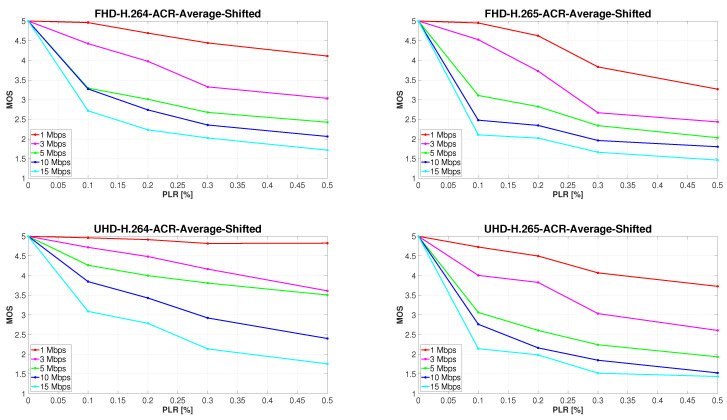
Development of subjectively perceived video quality with increasing PLR. MOS values were shifted in order to regard each video sequence unaffected by PLR as a reference.

In order to calculate and investigate the correlation between subjective and objective evaluation results, the MOS values were shifted in a way such that the highest score of the given sequence was always 5 (Figure 5), and thus the video unaffected by PLR was considered as a hidden reference. According to [30] the Pearson linear correlation coefficient was once more used to calculate the correlation.

Table 6 and Table 7 show the correlation between the results of subjective and objective quality assessment. The graphical analysis indicated that the main difference between these results is in the steepness of the decrease in video quality at different bit rates between 0 and 0.1% PLR. Therefore, the correlation of subjectively and objectively measured quality of only those video sequences that were affected by PLR is illustrated in Table 7. By removing sequences impaired solely by compression from the equation, the correlation of the results increased significantly. Nevertheless, it is apparent from Figure 5 and Table 6 and Table 7 that the correlation between subjective and objective assessment results is strong for all combinations of compression parameters and resolutions, with the lowest value of correlation coefficient of 0.83, calculated for H.265 ultra HD sequences at 15 Mbps. The observable differences are due to the fact that, unlike the algorithm, a person cannot separate the influence of PLR from the impact of bit rate on the visual quality of the evaluated video sequence. For this reason, a more detailed analysis of the combined effect of PLR and bit rate on the subjectively perceived video quality is presented in the following section.

### 5.3. Combined Impact of PLR and Compression on Subjective Video Quality

**Figure 6:**
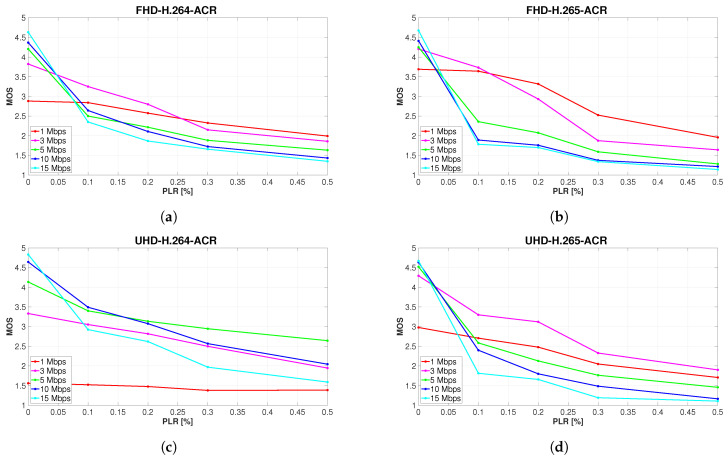
Development of subjectively perceived video quality with increasing PLR.

Although this paper focuses mainly on the impact of PLR, the subjective method used made it possible to further examine the mixed impact of PLR and compression on subjectively perceived video quality. To illustrate this combined effect, it was sufficient to leave the MOS values unchanged in the graphical representation of the results. This can be seen in Figure 6, which shows the overall picture of the development of perceived video quality affected by alteration of the compression parameters and the gradual increase in PLR. Each graph displays the average MOS values over all four types of sequences with different content. On the *x*-axes, PLR within the narrowed limits 0–0.5% is plotted, and the *y*-axes represent the measure of video quality on the five-level MOS scale.

A common aspect of Figure 6a–d is the inclination of perceived video quality at all bit rates to decline with the increasing PLR in correlation with the SSIM ratings. Another anticipated outcome is the increase of the MOS values of video sequences unaffected by packet loss as the bit rate rises. This trend is distinctly observable in the order of the individual curves representing the different bit rates at 0% PLR. However, it is apparent from the graphs that this arrangement changes substantially even if only 0.1% of packets are lost during video transmission. In fact, it follows that the subjective quality of video sequences affected by PLR also decreases with a steeper slope when a higher bit rate is transmitted. This was predicted from the found high correlation between subjective and objective evaluation results and can be seen in better clarity in Figure 5. Figure 6a,b reveal that the results of subjective quality evaluation of full HD sequences are highly consistent, regardless of the codec used. The main common characteristic of the assessment results of all videos at this resolution is the overall MOS distribution of the individual bit rates. It is worth noting that starting at 0.3% PLR, the quality ratings are exactly in the reverse order to the ratings of sequences unimpaired by packet loss.

**Figure 7:**
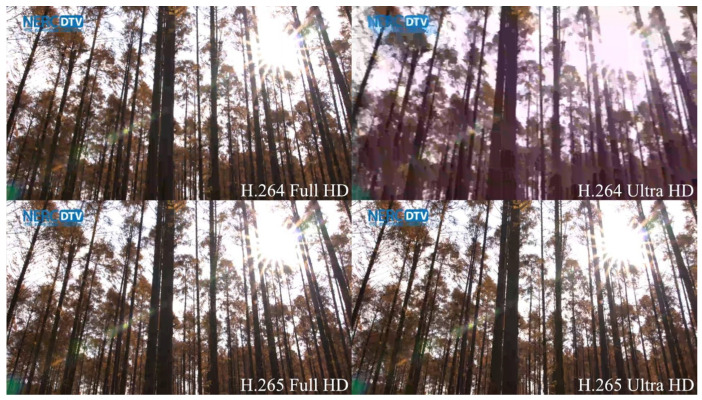
Preview of 1 Mbps Wood sequence in all examined compression formats and resolutions.

The essential distinction between the evaluation results of H.264 and H.265-encoded videos is the significantly more pronounced gap between the rating of lower bit rates (1 and 3 Mbps) compared to videos encoded at higher bit rates (5, 10, and 15 Mbps), which is evident on H.265 full HD sequences. This phenomenon is most eloquent when the simulated PLR reaches values from 0.1 to 0.2%. Another difference is a steeper decline in H.265 video quality, observable primarily at 10 and 15 Mbps. Unlike full HD videos, ratings of H.264-encoded ultra HD sequences diverged significantly from those at H.265 format. The only common aspects of Figure 6c,d are those that are universal for all four charts. As can be seen in Figure 6c, the average MOS of H.264 sequences shows that respondents considered 10 Mbps videos to retain the highest quality, when 0.1% packets were discarded during transmission. At higher PLR, 5 Mbps video sequences achieved the best subjective score. Although 1 Mbps sequences were rated higher than expected in the majority of the cases (as shown in Figure 6a,b,d), the quality of each H.264 ultra HD video at this bit rate was considered poor or bad, regardless of the PLR. It is therefore clear that in this particular case, the effect of compression on video quality was more intrusive than the loss of visual information. There is a straightforward explanation for this phenomenon, supported by Figure 7. The quality of H.264 ultra HD sequences at low bit rates is worse than the quality of the corresponding Full HD videos because four times the amount of information is discarded during compression due to the higher resolution. This effect is less pronounced when employing the H.265 codec, as HEVC compression is approximately 40% more efficient than older AVC [31].

Figure 6d indicates that the subjective MOS of H.265 videos affected to any extent by packet loss was unquestionably the highest when using a 3 Mbps bitrate.

### 5.4. Recommended Compression Parameters under Various Network Conditions

**Figure 8:**
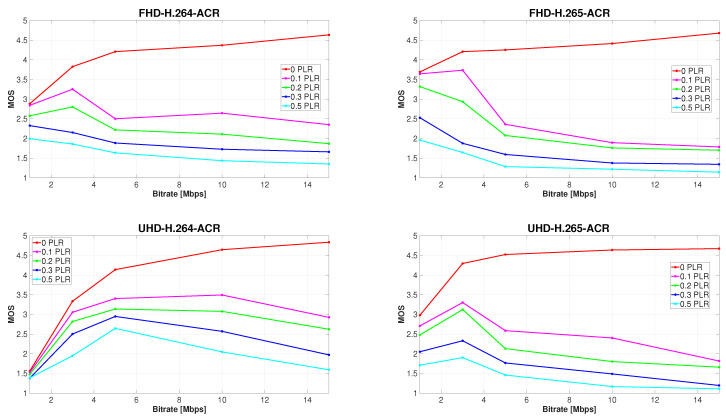
Development of subjectively perceived video quality with increasing bit rate.

If a certain packet loss rate in an error-prone network is anticipated, it may be convenient to get a better overview of how subjective quality changes as the bit rate increases or when other compression parameters are altered. For this reason, another four graphs were generated (Figure 8), with bit rate plotted on the *x*-axes. Each individual color curve represents a specific PLR. It is evident from these charts that subjective MOS values rise only until a certain value of bit rate is reached, and typically only at low PLR.

Based on the subjective data analysis, it is viable to determine the most suitable combination of resolution, bit rate, and codec for use under different conditions. If the video content is transmitted over a reliable network, the only variable is the adverse effect of compression. In such a case, deploying the higher bit rate below a certain threshold will most certainly increase the perceived video quality. However, after exceeding this limit, respondents may be unable to distinguish between the quality of two video sequences at consecutive data rates, which is why the differences in ratings are narrowing with increasing bit rate. The highest average MOS of 4.833 was achieved by H.264 ultra HD sequences at 15 Mbps. Video sequences at both resolutions and codecs were considered better than good (4 on the ACR scale) at 5, 10, and 15Mbps. By using the H.265 coding format, even 3 Mbps videos surpassed this value. Our research shows that if the assumed PLR in the network is less than 0.1%, it is best to encode the video to H.265 at full HD resolution at 3 Mbps. The average perceived quality of such sequences reached the MOS value of 3.733. The advantage is that the transmission of video with these specifications requires almost the narrowest bandwidth, and is therefore also the fastest and most economical. Although the average MOS of 1 Mbps videos with the same parameters is only slightly lower with a value of 3.642, employing this bitrate is not advisable due to the substantial disparity between ratings of sequences with different content, which we observed during the experiments. For ultra HD video, the highest average MOS of 3.492 was reached by H.264 sequences at 10 Mbps, closely followed by MOS 3.402 of videos at 5 Mbps. If the connection is extremely unreliable with a possible PLR of 0.2% or more, we recommend using an H.264 coding format, ultra HD resolution and 5 Mbps data rate, as the development of video quality with such parameters is the most constant, starting at MOS 3.136 and ending at 2.646 when packet loss reaches 0.5%. For full HD video, the H.265 codec at 1 Mbps seems to be the most advantageous, as the MOS starts at 3.317, although it drops sharply afterward to 1.958 at 0.5% PLR. For greater clarity, Table 8 lists the recommended bit rates for use with different combinations of compression parameters and expected PLR, based on subjective MOS.

## 6. Conclusions and Further Discussion

The aim of this paper was to analyze the impact of PLR on quality of video in full HD and ultra HD resolution encoded to H.264 and H.265 format at a bit rate ranging from 1 to 15 Mbps. First, a dataset of 11,200 test video sequences with eight different types of content combining all the abovementioned parameters and simulated PLR in range of 0 to 1% was compiled. Section 3 described the selection of sequences and their encoding. Subsequently, objective video quality evaluation was conducted by employing PSNR and SSIM metrics. Subjective quality assessment of 560 selected video sequences was performed by 36 laymen, using the popular ACR method. Evaluation of the video and analysis of the results were described in Section 4 by objective evaluation methods and in Section 5 by subjective evaluation methods. A high correlation was found between the results of evaluation methods; however, the subjective MOS revealed more about the mixed effect of PLR and compression.

Based on the analysis of the results, several conclusions were drawn. As was expected, the video quality declined along with the increasing PLR, irrespective of resolution, codec, or bitrate used. In case of video unaffected by packet loss, both measured and perceived quality improved with rising bit rate. However, this did not apply to video sequences impaired by PLR. In fact, it appears that in the presence of PLR, video quality decreases with a steeper slope when a higher data rate is transmitted. This is caused by the fact that use of a higher bit rate leads to a larger file size of the video sequence to be streamed over the error-prone network, thus splitting the image information into more UDP packets. At the same PLR, more of these packets are discarded during transmission which results in more individual or clustered losses that can propagate in time and space, causing further video quality degradation. It stemmed from the further analysis of subjective MOS that even 0.1% loss generally has more prominent negative effect on perceived video quality than H.264 or H.265 compression artifacts, which means that in most cases, increasing the bit rate over a given threshold value results in greater video-quality degradation. Based on this observation, the paper also includes a recommendation of compression parameters for use when various levels of PLR are expected. Packet losses may generate errors in different types of data. It depends on which part of the packet is affected by the loss. If the header of the packet is affected, which is the worst case, it is not possible to decode the whole packet. However, our effort was not to analyze in detail which part of the packet is influenced by the loss because in real traffic it is not able to control which part of the packet is affected by the loss. Our effort was to take an average in the loss rate, which we achieved by multiple transmission sequences.

## Figures and Tables

**Table 1:** The differences between the concepts of QoE and QoS.

Distinguishing Factors	QoS	QoE
Scope	Typically, telecom services	Broader domain, (not necessarily network-based)
Focus	Performance aspects of physical systems	Users’ assessment of system performance
Method	Technology oriented, empirical, or simulation measurements	Multi-disciplinary and multi-methodological approach

**Table 2:** Common parameters of reference sequences.

Resolution	Bit Depth	Aspect Ratio	Chroma Subsampling	Framerate [fps]	Length [s]
3840 × 2160 Ultra HD	10 bits per channel	16:9	4:4:4	30	10

**Table 3:** Description of codecs settings.

Command Example	Command Explanation
ffmpeg	run ffmpeg
-i Marathon_1920x1080_30fps_420_8bit_YUV.yuv	set the input file (name and container/uncompressed mode)
-vcodec libx264	set the codec
-x264-paramas	set the detailed H.264/AVC codec parameters
keyint=15	set the GOP length
min-keyint=15	set the minimum GOP length
bframes=3	set the number of B frames
b-adapt=1	disable the adaptivity of the B frames (default mode)
bitrate=1M	set the target bitrate
vbv-maxrate=1M	set the maxmum bitrate (by variable bitrate mode)
vbv-bufsize=1M	set the maximum buffer size
Marathon_1920x1080_30fps_420_8bit_H264_1M.ts	set the output file (name and container)

**Table 4:** Variable parameters of test sequences.

Parameter	Value
Codec	H.264 AVC, H.265 HEVC
Resolution	1920 × 1080 Full HD, 3840 × 2160 Ultra HD
Bitrate [Mbps]	1, 3, 5, 10, 15
Packet Loss Rate [%]	0, 0.1, 0.2, 0.3, 0.5, 0.75, 0.1

**Table 5:** File sizes of the H.265 UHD Marathon video sequence at 1–15 Mbps.

Test Sequence Name	File Size of the Test Sequence (in Bytes)
Marathon_UHD_30fps_420_8bit_H265_1M	1,436,508
Marathon_UHD_30fps_420_8bit_H265_3M	4,052,716
Marathon_UHD_30fps_420_8bit_H265_5M	6,784,544
Marathon_UHD_30fps_420_8bit_H265_10M	13,445,008
Marathon_UHD_30fps_420_8bit_H265_15M	20,205,864

**Table 6:** Correlation between the results of subjective and objective video quality evaluation.

PLCC—Lossless Video Sequences Included					
	**1 Mbps**	**3 Mbps**	**5 Mbps**	**10 Mbps**	**15 Mbps**
FHD—H.264	0.99	0.97	0.86	0.89	0.84
FHD—H.265	0.97	0.95	0.88	0.84	0.84
UHD—H.264	0.92	1.00	0.94	0.97	0.93
UHD—H.265	0.98	0.97	0.87	0.88	0.83

**Table 7:** Correlation between the results of subjective and objective video quality evaluation after removing lossless sequences.

PLCC—Lossless Video Sequences Excluded					
	**1 Mbps**	**3 Mbps**	**5 Mbps**	**10 Mbps**	**15 Mbps**
FHD—H.264	0.99	0.96	0.97	0.95	0.96
FHD—H.265	0.97	0.92	0.98	0.95	0.98
UHD—H.264	0.88	1.00	0.98	0.99	0.98
UHD—H.265	0.99	0.98	0.96	0.94	0.93

**Table 8:** Bit rate values at which subjective video quality is highest.

Expected PLR [%]		0	0.1	0.2	0.3	0.5
		Recommended Bitrate Based on MOS [Mbps]				
Full HD	H.264	15	3	3	1	1
	H.265	15	3	1	1	1
Ultra HD	H.264	15	10	5	5	5
	H.265	15	3	3	3	3

## Data Availability

Data available on request from the authors.
